# Robust Adaptive Immunity to MPXV in Older People Who Received Childhood Vaccinia Vaccination

**DOI:** 10.3390/biology15030234

**Published:** 2026-01-26

**Authors:** Chris Davis, Jianmin Zuo, Rachel Bruton, Marie Hodges, Tom Roberts, Maria Manali, Paula Olmo, Brian Willett, Paul Moss, Helen Parry

**Affiliations:** 1 MRC University of Glasgow Centre for Virus Research, University of Glasgow, Glasgow G61 1QH, UK; maria.manali@glasgow.ac.uk (M.M.); paula.olmo@glasgow.ac.uk (P.O.); brian.willett@glasgow.ac.uk (B.W.); 2 Department of Immunology and Immunotherapy, School of Infection, Inflammation and Immunology, College of Medicine and Health, University of Birmingham, Birmingham B15 2SY, UK; j.zuo@bham.ac.uk (J.Z.); r.k.bruton@bham.ac.uk (R.B.); m.hodges.1@bham.ac.uk (M.H.); tom.roberts@bioch.ox.ac.uk (T.R.); p.moss@bham.ac.uk (P.M.); 3 Centre for Clinical Haematology, University Hospitals Birmingham, Edgbaston, Birmingham B15 2SY, UK

**Keywords:** monkeypox virus, historical vaccination, immune responses

## Abstract

Monkeypox virus (MPXV) is a zoonotic *Orthopoxvirus* responsible for Monkeypox (Mpox), historically associated with sporadic zoonotic transmission but increasingly characterised by sustained human-to-human spread. While vaccinia-based vaccination is known to confer cross-protection against MPXV, the durability of such immunity over a human lifetime remains incompletely characterised. Here, we assessed humoral and cellular immune responses to MPXV in octogenarians and nonagenarians vaccinated against smallpox during childhood. Twenty-three adults aged 79–94 years (median 83), who self-reported childhood vaccinia vaccination between 1925 and 1940, were recruited. MPXV-specific antibody responses were evaluated using ELISA, targeting homologous vaccinia and MPXV proteins, and live-virus neutralisation assays. Cellular immunity was assessed by IFN-γ ELISpot following stimulation with peptide pools derived from highly conserved vaccinia antigens. Responses were also obtained from younger, recently MVA–BN-vaccinated and unvaccinated control donors. All historically vaccinated participants exhibited MPXV-reactive IgG responses, with antibody binding and neutralisation levels comparable to recently vaccinated individuals. Functional neutralising activity against MPXV was detected in all donors, with ≥50% neutralisation observed in 78% of participants. Antibody concentrations correlated strongly with neutralisation capacity. T-cell responses were detectable in all historically vaccinated donors, most prominently against the major core protein A10L, although reduced magnitudes were observed in participants over 90 years of age. No MPXV-specific humoral or cellular responses were detected in unvaccinated controls. These findings demonstrate that childhood vaccinia vaccination induces durable humoral and cellular immunity against MPXV persisting for over seven decades. Historical smallpox vaccination status may therefore remain a relevant determinant of protection against Mpox.

## Simple Summary

Vaccination against smallpox provides protection from Monkeypox virus, which has recently shown an increase in cases. We assessed adults aged 79–94 years who were vaccinated against smallpox as children and showed both antibody and T-cell memory responses against Monkeypox virus. No such responses were found in unvaccinated individuals. This suggests that receiving the smallpox vaccination as a child can provide long-lasting protection against Monkeypox, decades later.

## 1. Introduction

Monkeypox virus (MPXV), the causative agent of Mpox, is a zoonotic *Orthopoxvirus* first described in 1958 and endemic to central and West Africa. Historically, MPXV has caused outbreaks due to zoonotic spillover events, with sporadic cases recorded increasingly in non-endemic countries over the past two decades, arising mostly from human travel [1].

However, in 2022, the World Health Organization reported a large global outbreak of sustained human-to-human transmission of Clade IIb MPXV. As of 31 October 2025, 168,736 confirmed cases of Mpox had been reported globally from 142 countries and territories [2]. Whilst Clade IIb peaked outside of Africa, a newly designated Clade Ib MPXV was declared a public health emergency of international concern on 14th August 2024, with confirmed cases in Europe, Asia, and the Americas, although the incidence remains substantially higher in endemic African settings as well as neighbouring African countries. The current situation in the DRC is the concurrent circulation of both Clades Ia and Ib, leading to a significant number of confirmed cases, with >35,000 cases between January 2024 and October 2025, and a larger number of suspected cases remaining untested [3].

Widespread delivery of the vaccinia vaccine underpinned the achievement of global eradication of smallpox (variola) in 1980. However, routine vaccinia delivery was discontinued in many countries by the early 1970s, as effective herd immunity was achieved at the population level. In the UK, mandated smallpox vaccination ceased in 1948, with the programme ending completely in 1971 [4]. The vaccine used in the UK prior to 1971 was derived from the Lister strain [5].

Strong immunological cross-protection against Mpox is achieved following vaccinia vaccination and has proven to be the most effective way of preventing outbreaks and dissemination of Mpox. The Modified Vaccinia Ankara–Bavarian Nordic (MVA–BN) vaccine is now offered as post-exposure prophylaxis to close contacts of index cases, as well as for vaccination campaigns among at-risk populations (including men who have sex with men). At the population level, 50 years has passed since the cessation of the vaccination campaign against smallpox. As a result, waning immunity against *Orthopoxviruses* and changing population demographics are likely accountable for the increasing sporadic outbreaks that have occurred over the past two decades, raising the potential for MPXV vaccine escape to arise.

Vaccine-conferred immunity arising from childhood smallpox vaccination may be adequately sustained such that protection from Mpox is achieved in the elderly, although immunosenescence may attenuate antigen-specific memory recall. Recent reports indicate that humoral immunity, manifesting as MPXV-specific neutralisation, is retained in individuals born between 1919 and 1925 who were vaccinated as children [6]. Cellular immune recognition of *Orthopoxviruses* is important in the control of disease dissemination. The longevity of such immunity following smallpox vaccination has been demonstrated in individuals whose births date to around 1945 [7]. To expand this data further, we determined humoral and T-cell immunity against *Orthopoxviruses* in elderly individuals who received vaccinia vaccination between 1925 and 1940. The median age of the 23 individuals recruited to the study group was 83 years (IQR 81–84) and 15/23 were female (65%). Blood samples were also taken from two donors aged 38 and 40 years at 4 weeks following single-dose vaccination with Ankara–Bavarian Nordic (MVA–BN), and two unvaccinated donors aged 23 and 41 years for control purposes.

## 2. Materials and Methods

### 2.1. Patient Cohort

Participants were recruited through a primary care network in the West Midlands, UK, between 29 December 2020 and 28 February 2021, as part of the UK Coronavirus Immunological Analysis and emerging virus work [8]. Individuals living independently aged ≥79 were identified from primary care records and invited by letter to take part. Those wishing to take part contacted the study co-ordinator and provided written informed consent. A total of 103 participants were re-approached in September 2023 prior to receiving the Autumn vaccination programme dose and as part of ongoing surveillance for the cohort. At this pre-vaccine visit, participants were asked if they recalled being vaccinated against smallpox as a child and a blood sample was taken. In total, 23/103 donors aged between 79 and 94 years old confirmed they had memory of being vaccinated against smallpox as children. A further four individuals were recruited within the University of Birmingham, two of whom had healthcare professional roles and had been vaccinated 4 weeks prior with the Ankara–Bavarian Nordic (MVA–BN) vaccine as part of post-exposure prophylaxis, and two unvaccinated individuals. Details of the ages and status of each donor can be found in Table 1. Participants provided written consent, while ethical approval was obtained from The North West Preston Research Ethics Committee with a favourable outcome (number REC 20/NW/0240). The study was undertaken according to the Declaration of Helsinki and Good Clinical Practice guidelines.

### 2.2. Sample Preparation

In total, 50 mL of blood was obtained by phlebotomy. Serology samples were collected in BD serum separating tubes and used for serological testing. Peripheral blood mononuclear cells (PBMCs) for cellular assays were prepared from blood collected in lithium heparin tubes and processed within 24 h of phlebotomy. Following 50:50 dilution with Roswell Park Memorial Institute (RPMI) medium, PBMCs were isolated on a Sepmate Ficoll density gradient (Stemcell, Cambridge, UK), washed with RPMI, and frozen in foetal bovine serum (FBS) with 10% Dimethyl sulfoxide (DMSO) (Sigma-Aldrich, Gillingham, UK).

### 2.3. Virus Stock

Clinical MPXV CVR_S1 was isolated from an ISARIC4C patient by plating on Vero cells (ATCC; CRL-1586). Ethical approval for the ISARIC CCP-UK study was given by the South Central–Oxford C Research Ethics Committee in England (13/SC/0149), the Scotland A Research Ethics Committee (20/SS/0028) and the WHO Ethics Review Committee (RPC571 and RPC572). The virus was subjected to Illumina metagenomic sequencing to evaluate purity and sequence homology to pre-culture clinical material (CVR_MPXV1a; accession number ON808413, Clade IIb) to confirm the sequence identity and source of the propagated isolate. All experiments were performed in a biosafety level 3 (BSL-3) laboratory at the MRC-University of Glasgow Centre for Virus Research (SAPO/223/2017/1a). Virus stock concentrations were determined by plaque formation assays. In brief, Vero cells were seeded at 2 × 10^5^ cells per well 24 h prior to inoculation. The virus stock was serially diluted 1:10, and 200 uL of these dilutions was added to the cells and allowed to infect for one hour before the addition of 1 mL DMEM containing 10% FBS. The plates were incubated for 48 h at 37 °C before fixing in 8% (*v*/*v*) formaldehyde (Sigma-Aldrich, F8775), washed in phosphate-buffered solution (PBS), and stained with 0.1% Coomassie Brilliant Blue solution (Sigma-Aldrich, B0149) in 45% methanol and 10% acetic acid for up to 30 min. Plates were washed in water and dried overnight before plaque counting under a plate microscope and calculation of the plaque-forming units/mL (PFU) of the stock.

### 2.4. ELISA

MPXV stocks were diluted in PBS and used to coat an Immulon 2HB 96-well flat-bottom plate (Thermo Fisher Scientific, Altrincham, UK) at a concentration of 1  ×  10^5^ pfu per well. The plates were incubated on an orbital shaker overnight at 4 °C. Plates were then blocked with PBS-T (PBS with 0.1% (*v*/*v* Tween-20) containing 5% bovine serum albumen (BSA) for one hour before the addition of the samples. The plates were washed five times in PBS-T before the serum samples were added (diluted 1:50 in PBS in triplicate, as set out by the National Institute of Biological Standards and Control, UK for MPXV) [9]. Samples were incubated at room temperature on an orbital shaker for one hour before washing five times in PBS-T and adding anti-human IgG (Merck, Darmstadt, Germany, A1543) diluted 1:10,000. The plates were incubated for a further hour before washing five times with PBS-T and then 100 µL of alkaline phosphatase yellow (Merck, P7998), and incubated for 15 minutes before stopping the reaction with 100 µL 3 M sodium hydroxide (NaOH). The absorbance was measured at 405 nm. Samples were determined as positive if the absorbance value was above 2x the mean of background wells.

### 2.5. Electrochemiluminescence Assay

IgG antibodies against VACV and MPXV were quantified by electrochemiluminescence (ECL) assays using the Meso Scale Discovery (MSD) V-PLEX *Orthopoxvirus* Panel 1 (IgG) kit (Catalogue # K156882U). Each well contained 10 spots: five coated with VACV proteins (A27L, D8L, L1R, A33R and B5R), and five coated with MPXV proteins (A29L, E8L, M1R, A35R and B6R). MSD ECL assays were performed according to the manufacturer’s instructions. Briefly, samples were diluted (1:500) and added to plates that had been blocked, along with calibrator and control samples. Plates were incubated for two hours and then washed. SULF-TAG-conjugated anti-human IgG was then added, and the plates were incubated for one hour. After incubation, plates were washed and read using a MESO Sector S 600 plate reader (Meso Scale Diagnostics, London, UK). Electrochemiluminescence data were processed using Methodological Mind software (version 4.0) and analysed using MSD Discovery Workbench (version 4.0). Results were expressed as MSD arbitrary units per mL (AU/mL).

### 2.6. Neutralisation 

Vero cells were seeded at a concentration of 1 × 10^4^ cells per well in a 96-well plate for 24 h prior to inoculation. Virus stocks were diluted to 50 plaque-forming units (pfu) and mixed 1:1 with heat-inactivated patient serum diluted 1:25 in DMEM, 10% FBS, to yield a final concentration of 25 pfu and 1:50 serum dilution. This mixture was incubated for one hour at 37 °C before being applied to 96-well plates in triplicate. The plates were incubated for 48 h before fixing in 8% (*v*/*v*) formaldehyde (Sigma-Aldrich, F8775), washed in PBS, and stained with 0.1% Coomassie Brilliant Blue solution (Sigma-Aldrich, B0149) in 45% methanol and 10% acetic acid for up to 30 min. Plates were washed in water and dried overnight before plaque counting under a plate microscope. The mean plaque count was then compared to the untreated infected wells to yield a percentage neutralisation.

### 2.7. ELISpot

T-cell responses were measured using an IFN-γ ELISpot Pro kit (Mabtech, Stockholm, Sweden) following PBMC stimulation with 4 differing peptide pools of 15 mers with 11-amino-acid overlap (JPT, Berlin, Germany). Pools 1 and 2 consisted of 220 peptides from major core protein A10L (MVA 121L) of vaccinia virus (with 98.3% homology to MPXV A11L) (Swiss-Prot ID: O57223) and comprised 110 peptides in each of 2 vials. Pool 3 consisted of 35 peptides derived from a peptide scan through host range protein 2 C7L (MVA018L) of the VACV-Ankara strain, with 99.3% homology with MPXV D10L (Swiss-Prot ID: P68598). Pool 4 comprised 74 peptides derived from a peptide scan through cell surface-binding protein D8L (MVA105L) of VACV Ankara strain with 97% homology to MPXV E8L (Swiss-Prot ID: O57211).

PBMCs were defrosted and rested overnight and 0.25  ×  10^6^ cells added in duplicate to wells containing each peptide pool at a final individual peptide concentration of 1 μg/mL, or to dimethyl sulphoxide-only (negative) control, or to a single well containing mitogenic anti-CD3 antibody (positive control). Samples were incubated for 16–18 h, and ELISpot plates were developed following the manufacturer’s instructions and read using an AID plate reader (AID, Strassberg, Germany). Values were normalised by subtraction of the negative-control wells and results are presented as spot-forming cells per million PBMCs.

## 3. Results

**Figure 1:**
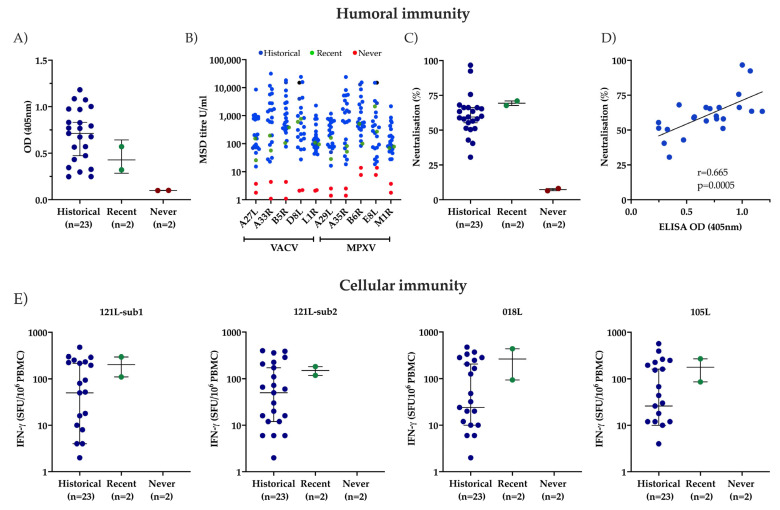
Humoral and cellular immune responses to MPXV by vaccinia vaccination status. Elderly participants with a history of childhood vaccinia vaccination (blue dots), young control participants recently vaccinated with vaccinia (green dots) and participants never vaccinated (red dots) are shown. (**A**) represents a dot plot of MPXV-specific antibody responses to whole virus measured by ELISA (median and 95% CI shown). (**B**) demonstrates the antibody-specific responses assessed using the MSD platform to VACV and MPXV protein homologues (A27L-A29L; A33R-A35R; B5R-B6R; D8L-B6R and L1R-M1R). (**C**) Live MPXV neutralisation data is represented in a dot plot with medians and 95% CIs shown. (**D**) The correlation between ELISA-derived MPXV-specific antibody titres and MPXV neutralisation is shown as determined using Spearman’s rank correlation coefficient r = 0.66; *p* = 0.0005). (**E**) ELISpot results showing the number of spot-forming units/10^6^ PBMCS following peptide pool stimulations against 121 L, 018 L and 105 L and by vaccination status (historical, recently or never vaccinated) are demonstrated, with the median and interquartile ranges represented for each group.

Cross-reactive antibody responses to MPXV were first assessed using ELISA and were detectable in all elderly participants and recent MVA-BN-vaccinated donors (Figure 1A). No response was observed in unvaccinated donors (median OD value of historically vaccinated (*n* = 23): 0.71; range 0.24–1.18). Using the MSD platform, antibody responses to five proteins specific to VACV or MPXV were next investigated. Here, no difference was observed in antibody responses amongst elderly, historically vaccinated donors for VACV (median 399.5) and MPXV proteins (MPXV 400) (*n* = 21 *p* = 0.36 (Mann–Whitney), whilst those unvaccinated had no detectable responses to either (Figure 1B).

**Figure 2:**
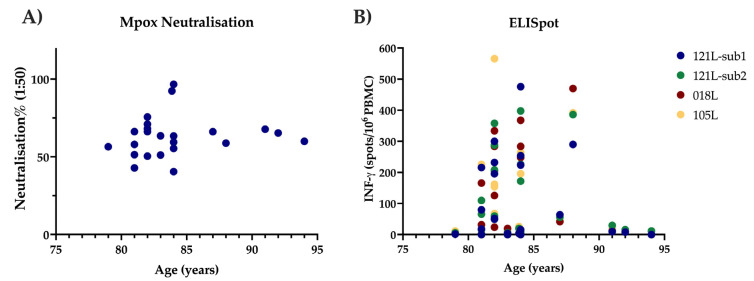
Relationship of immune response to participant age. (**A**) Neutralisation responses to live MPXV are demonstrated according to the age at the time of sampling of participants who received historical vaccinia vaccination during childhood. (**B**) ELISPOT responses to 4 different peptide pools (121 L-sub1, 121 L-sub2, 018 L and 105 L) with close homology to MPXV are shown in those historically vaccinated with vaccinia in childhood and according to their age at the time of sampling.

Next, neutralising activity against MPXV was assessed. All elderly vaccinated donors had detectable functional responses, with 18/23 participants demonstrating a neutralisation level of 50% or more (Figure 1C). Indeed, no negative impact of age on antibody response was observed, and three nonagenarian participants had mean neutralisation percentages of 67.7%, 65.2% and 60% against MPXV. Values of 67.7% and 71% were observed in the two younger control participants who recently received MVA-BN (Figure 2A). Antibody concentration and neutralising activity were strongly correlated (Spearman’s rank correlation coefficient r = 0.66; *p* = 0.0005) (Figure 1D).

Cellular immunity was assessed by ELISpot analysis against peptide pools for (1) the major core protein A10L of VACV, (2) host range protein 2 C7L, and (3) the cell surface-binding protein D8L. Positive control values, elicited from mitogenic CD3-specific antibodies, were seen in all donors and comparable.

The greatest cellular responses were observed against major core protein A10L, with a median response in historically vaccinated participants of 51 SFU/10^6^ PBMC (IQR 4-222) (sub1) and 53 SFU/10^6^ PBMC (IQR 12-199) (sub 2). Values of 110 and 296 SFU/106 (sub 1) and 118 and 182 SFU/10^6^ PBMC (sub 2) were observed in the two recently vaccinated donors. The median response to C7L was 28 SFU/10^6^ PBMC (IQR 7-237.5) and 22 SFU/10^6^ PBMC (IQR 5-187.5) for D8L, compared to 94 and 86 SFU/10^6^ PBMC for control participant 1 and 434 and 270 SFU/10^6^ PBMC for control participant 2, respectively. All historically vaccinated donors had a response of >10 SFU per million PBMCs to at least one individual peptide pool tested. No clear association was seen between donor age and magnitude of MVA-specific immune response, although a trend towards lower cellular responses was observed in nonagenarians, where only those against the major core protein A10L were detectable (Figure 2B).

## 4. Discussion

In this study, we examined humoral and cellular immunity against MPXV in a cohort of 23 elderly donors who self-reported receiving vaccinia vaccination during childhood. Strikingly, despite a period of 70–80 years since vaccination, humoral immunity against MPXV and cellular responses to peptides from VACV with high homology to MPXV, were observed in all donors. These results indicate that childhood vaccinia vaccination leads to a robust and lifelong adaptive immune response against homologous *Orthopoxvirus* [6,10,11,12]. Previous work has also reported stable antibody responses for decades following vaccinia vaccination, and together these findings reveal an extreme resilience of the humoral immune response to *Orthopoxvirus.* Prior studies have indicated that virus-specific T-cell responses have a half-life of between 8 and 12 years, and reliable detection requires ex vivo expansion in some cases [10,12]. Our findings demonstrate that intact cellular immunity was present in all donors without the requirement of expansion. This ex vivo detection augurs well for cell expansion following an *Orthopoxvirus* infection. Some of these differences may derive from the nature of the vaccine immunogen, where prior studies examined the responses to Tian tan strain challenge in childhood compared to the Lister strain that was administered in this UK cohort.

Immune senescence is a potentially important determinant of immune protection, and we did observe an attenuated cellular response in donors over 90 years of age, which may reflect the contracted T-cell pool, with reduced T-cell diversity and frequency observed with natural age-associated waning of immunity. Nevertheless, cellular immunity was detectable at a low level in all donors, with >10 SFC/10^6^ PBMCs observed against at least one peptide pool tested (Table 1). Whilst it is not clear if a threshold of 10 SFU/10^6^ viruses provides sterilising immunity, a detectable T-cell response to an individual peptide pool is likely to contribute to rapid memory T-cell expansion and disease mitigation through recognition and elimination of infected cells, thus reducing the infectious period.

The findings from this study further emphasise the importance of increased understanding of the immunological memory induced by vaccinia vaccination, especially considering the ongoing threat posed by Mpox. This is of note in relation to cellular immunity as *Orthopoxvirus*-recognising T-cells are essential for controlling viral replication and clearance of infected cells, as well as providing support for antibody production. An absence of IFN-γ production is associated with a greater lethality of Mpox in animal models [13]. Furthermore, recent studies have shown that patients with uncontrolled HIV infection may develop a fulminant form of Mpox, further highlighting the requirement of a robust adaptive immune response [14,15].

Limitations of our study include the potential bias within the recruitment process, as this was reliant on participants’ self-reported childhood smallpox vaccination and approximate dates, limiting accuracy. The clinical histories of participants’ exposure to smallpox or other *Orthopoxviruses* were not accessible, which may have boosted existing vaccine-induced immunity. During the period when the participants reported receiving the vaccine, the UK had a mandatory vaccine policy for children, under the Vaccination Act. 1907 [16], although conscientious objector certificates could be obtained. It is however important to note that endemic smallpox in the UK was eradicated following the Variola minor epidemic from 1920 to 1935, which resulted in 81,983 cases across England and Wales, with only minor imported cases seeding small epidemics (totalling around 569 cases across the UK) between 1936 and 1970 [4,17]. Other *Orthopoxviruses* would be capable of producing a cross-reactive immune response, but only Cowpox (CPXV) is known to be endemically present in the UK. CPXV is maintained in rodent reservoirs, wood mice, bank voles, and short-tailed field voles [18]. Sporadic outbreaks of CPXV can occur in domestic animals, such as cats, dogs and horses, which in turn, can lead to human transmission. But this is extremely rare and only in the order of 1–2 cases per year [19,20,21]. Given the low number of cases of both smallpox and CPXV cases in the UK since the 1940s, and considering that the smallpox vaccine was 95% effective in preventing infection, the robust adaptive immunity observed in participants is most likely due to the vaccination rather than a boosting effect from post-vaccination exposure. It is also worth noting that the samples were collected before 2022, making recent exposure to MPXV unlikely.

Another potential limitation relates to the relatively small sample size for this study; access to participant cohorts within this age range (median age 83) is challenging. Additionally, identifying appropriate age-matched controls for vaccination proved unattainable due to the cessation of mandated vaccination in the UK since the 1950s. The small number of recently vaccinated control participants reflects the selective nature of the current vaccination programme, which is limited to individuals at high risk of exposure. Therefore, any comparison between the control group and the participants will be confounded by differences in age and vaccination platform. Additional comparative work with greater numbers of younger, vaccinated individuals is now needed in order to understand whether the magnitude of cellular responses observed in the initial years following vaccination differs from our observed responses in the elderly, historically vaccinated.

The choice of peptides used for cellular assays within this work reflected the availability at the time the experimental work took place. Vaccinia-based peptides have 97–99% homology to MPXV and almost certainly provide reactivity relevant to MPXV. Indeed, the homology far exceeds the ~67% amino acid homology provided as a benchmark for cross-reactivity [22], but additional important MPXV-specific responses that are not represented through the tested peptide pools may have been missed. As such, further work using MPXV-specific peptide pools which are now commercially available would be of interest to study and compare with our findings.

Our findings have implications for international public health management of Mpox. People who received vaccinia vaccination prior to the end of the smallpox eradication campaign are likely to harbour strong ongoing protection against severe Mpox. Indeed, the increase in cases of Mpox in the last 40 years may partially reflect cessation of the smallpox eradication programme and the potential creation of a vacated ecological niche [23]. The use of historical smallpox vaccination records could therefore be used to identify those members of the population at greatest risk and target vaccination programmes. This is of considerable importance given current limitations in vaccine supply [24,25]. Furthermore, whilst the SARS-CoV-2 pandemic revealed the potential extreme vulnerability of older people to a new virus, our data indicate that people within this age group retain strong adaptive immune responses over many decades following vaccinia challenge, and as such, do not represent a vulnerable group for Mpox if they received smallpox vaccination during childhood.

## 5. Conclusions

The persistence of T-cell and humoral responses to MPXV in octo- and nonagenarians vaccinated against smallpox highlights the long-term effectiveness data of the vaccinia vaccine and its role in providing cross-protection against other *Orthopoxviruses*. These findings support the consideration of historical smallpox vaccination status in current Mpox outbreak management strategies and suggest that the vaccinia vaccine-induced immunity may provide ongoing value against future *Orthopoxvirus* threats. Further work is now required to understand whether the change to the MVA-BN vaccine platform or the potential future adoptions of an mRNA-based vaccine will affect the longevity of the immunological responses compared to historical vaccinia vaccine strains. In addition, the MVA strain is being utilised as a vaccine platform to target pathogens other than *Orthopoxviruses* by creating recombinant viruses bearing the proteins of the target pathogen. These include the licensed vaccine for use against Ebola (Mvabea) [26], as well as vaccines against Influenza [27], Tuberculosis [28], Respiratory Syncytia Virus [29] and others undergoing clinical trials. Understanding the effects of this delivery method in terms of immunological longevity against *Orthopoxviruses* and the target organism will be of great interest.

## Figures and Tables

**Table 1:** Participant age and vaccination status.

Doner	Sex	Age (at Recruitment)	Vaccinated
1	F	83	Childhood
2	M	84	Childhood
3	F	79	Childhood
4	F	84	Childhood
5	F	82	Childhood
6	F	81	Childhood
7	F	88	Childhood
8	M	81	Childhood
9	F	82	Childhood
10	M	82	Childhood
11	M	82	Childhood
12	F	81	Childhood
13	F	83	Childhood
14	M	81	Childhood
15	f	83	Childhood
16	F	84	Childhood
17	M	91	Childhood
18	F	84	Childhood
19	m	84	Childhood
20	M	82	Childhood
21	F	94	Childhood
22	F	87	Childhood
23	M	92	Childhood
SP1	F	40	Adult
SP2	F	38	Adult
HC1	F	41	Unvaccinated
HC2	M	23	Unvaccinated

## Data Availability

All data is available within the publication.

## Abbreviations

The following abbreviations are used in this manuscript:

MPXV	Monkeypox virus
MSD	Meso Scale Discovery

## References

[1] Haider N., Guitian J., Simons D., Asogun D., Ansumana R., Honeyborne I., Velavan T.P., Ntoumi F., Valdoleiros S.R., Petersen E. (2022). Increased outbreaks of monkeypox highlight gaps in actual disease burden in Sub-Saharan Africa and in animal reservoirs. Int. J. Infect. Dis..

[2] https://www.paho.org/sites/default/files/2025-12/sitrep-mpox-nov-2025pdf.pdf.

[3] https://worldhealthorg.shinyapps.io/mpx_global/#sec-clades.

[4] Millward G. (2019). Vaccinating Britain: Mass Vaccination and the Public Since the Second World War [Internet]; Select bibliography.

[5] Parrino J., Graham B.S. (2006). Smallpox vaccines: Past, present, and future. J. Allergy Clin. Immunol..

[6] Dee K., Manali M., Bissett L.A., Bone J., Magill C., Davis C., Willett B.J., Murcia P.R. (2024). Smallpox vaccination campaigns resulted in age-associated population cross-immunity against monkeypox virus. J. Gen. Virol..

[7] Adamo S., Gao Y., Sekine T., Mily A., Wu J., Storgärd E., Westergren V., Filén F., Treutiger C.-J., Sandberg J.K. (2023). Memory profiles distinguish cross-reactive and virus-specific T cell immunity to Mpox. Cell Host Microbe.

[8] Parry H., Bruton R., Stephens C., Bentley C., Brown K., Amirthalingam G., Hallis B., Otter A., Zuo J., Moss P. (2022). Extended interval BNT162b2 vaccination enhances peak antibody generation. NPJ Vaccines.

[9] https://nibsc.org/documents/ifu/22-218.pdf.

[10] Hammarlund E., Lewis M., Hansen S., Strelow L.I., Nelson J.A., Sexton G.J., Hanifin J.M., Slifka M.K. (2003). Duration of antiviral immunity after smallpox vaccination. Nat. Med..

[11] Li E., Guo X., Hong D., Gong Q., Xie W., Li T., Wang J., Chuai X., Chiu S. (2023). Duration of humoral immunity from smallpox vaccination and its cross-reaction with Mpox virus. Signal Transduct. Target. Ther..

[12] Li M., Guo Y., Deng Y., Gao W., Huang B., Yao W., Zhao Y., Zhang Q., Huang M., Liu M. (2024). Long-lasting humoral and cellular memory immunity to vaccinia virus Tiantan provides pre-existing immunity against Mpox virus in Chinese population. Cell Rep..

[13] Earl P.L., Americo J.L., Moss B. (2012). Lethal Monkeypox Virus Infection of CAST/EiJ Mice Is Associated with a Deficient Gamma Interferon Response. J. Virol..

[14] Abdulkarim B., Ticknor I.L., Torres A.R., Mohammed T.O., Rees J.S., Baghchechi M., Streams B.N. (2023). Cutaneous findings of fulminant monkeypox in a patient with HIV/AIDS. JAAD Case Rep..

[15] Thornhill J.P. (2022). Monkeypox Virus Infection in Humans across 16 Countries. N. Engl. J. Med..

[16] https://www.legislation.gov.uk/ukpga/Edw7/7/31/enacted.

[17] Rafferty S., Smallman-Raynor M.R., Andrew D., Cliff A.D. (2018). Variola minor in England and Wales: The geographical course of a smallpox epidemic and the impediments to effective disease control, 1920–1935. J. Hist. Geogr..

[18] Chantrey J., Meyer H., Baxby D., Begon M., Bown K.J., Hazel S.M., Jones T., Montgomery W.I., Bennett M. (1999). Cowpox: Reservoir hosts and geographic range. Epidemiol. Infect..

[19] Costa T., Stidworthy M.F., Ehmann R., Denk D., Ashpole I., Drake G., Maciuca I., Zoeller G., Meyer H., Chantrey J. (2023). Cowpox in zoo and wild animals in the United Kingdom. J. Comp. Pathol..

[20] Lawn R. (2010). Risk of cowpox to small animal practitioners. Vet. Rec..

[21] Bruneau R.C., Tazi L., Rothenburg S. (2023). Cowpox Viruses: A Zoo Full of Viral Diversity and Lurking Threats. Biomolecules.

[22] Mateus J., Grifoni A., Tarke A., Sidney J., Ramirez S.I., Dan J.M., Burger Z.C., Rawlings S.A., Smith D.M., Phillips E. (2020). Selective and cross-reactive SARS-CoV-2 T cell epitopes in unexposed humans. Science.

[23] James O. (2013). Lloyd-Smith; Vacated niches, competitive release and the community ecology of pathogen eradication. Philos. Trans. R. Soc. Lond. B Biol. Sci..

[24] Moss B. (2011). Smallpox vaccines: Targets of protective immunity. Immunol. Rev..

[25] Rimoin A.W., Mulembakani P.M., Johnston S.C., Smith J.O.L., Kisalu N.K., Kinkela T.L., Blumberg S., Thomassen H.A., Pike B.L., Fair J.N. (2010). Major increase in human monkeypox incidence 30 years after smallpox vaccination campaigns cease in the Democratic Republic of Congo. Proc. Natl. Acad. Sci. USA.

[26] Milligan I.D., Gibani M.M., Sewell R., Clutterbuck E.A., Campbell D., Plested E., Nuthall E., Voysey M., Silva-Reyes L., McElrath M.J. (2016). Safety and Immunogenicity of Novel Adenovirus Type 26–and Modified Vaccinia Ankara–Vectored Ebola Vaccines: A Randomized Clinical Trial. JAMA.

[27] de Vries R.D., Altenburg A.F., Nieuwkoop N.J., de Bruin E., van Trierum S.E., Pronk M.R., Lamers M.M., Richard M., Nieuwenhuijse D.F., Koopmans M.P.G. (2018). Induction of Cross-Clade Antibody and T-Cell Responses by a Modified Vaccinia Virus Ankara–Based Influenza A(H5N1) Vaccine in a Randomized Phase 1/2a Clinical Trial. J. Infect. Dis..

[28] Tameris M.D., Hatherill M., Landry B.S., Scriba T.J., Snowden M.A., Lockhart S., Shea J.E., McClain J.B., Hussey G.D., Hanekom A.W. (2013). Safety and efficacy of MVA85A, a new tuberculosis vaccine, in infants previously vaccinated with BCG: A randomised, placebo-controlled phase 2b trial. Lancet.

[29] Jordan E., Lawrence S.J., Meyer T.P.H., Schmidt D., Schultz S., Mueller J., Stroukova D., Koenen B., Gruenert R., Silbernagl G. (2021). Broad Antibody and Cellular Immune Response from a Phase 2 Clinical Trial with a Novel Multivalent Poxvirus-Based Respiratory Syncytial Virus Vaccine. J. Infect. Dis..

